# Ultra‐low dose CT colonography with automatic tube current modulation and sinogram‐affirmed iterative reconstruction: Effects on radiation exposure and image quality

**DOI:** 10.1002/acm2.12510

**Published:** 2018-12-26

**Authors:** Roberta Cianci, Andrea Delli Pizzi, Gianluigi Esposito, Mauro Timpani, Alessandra Tavoletta, Pierluigi Pulsone, Raffaella Basilico, Antonio Raffaele Cotroneo, Antonella Filippone

**Affiliations:** ^1^ Department of Neuroscience Imaging and Clinical Sciences University “G. d'Annunzio”, SS. Annunziata Hospital Chieti Italy

**Keywords:** computed tomographic colonography, computed tomographic colonography, technique, radiation dose, dose reduction, automated tube current modulation, iterative reconstruction techniques

## Abstract

**Objective:**

To assess the radiation dose and image quality of ultra‐low dose (ULD)‐CT colonography (CTC) obtained with the combined use of automatic tube current (mAs) modulation with a quality reference mAs of 25 and sinogram‐affirmed iterative reconstruction (SAFIRE), compared to low‐dose (LD) CTC acquired with a quality reference mAs of 55 and reconstructed with filtered back projection (FBP).

**Methods:**

Eighty‐two patients underwent ULD‐CTC acquisition in prone position and LD‐CTC acquisition in supine position. Both ULD‐CTC and LD‐CTC protocols were compared in terms of radiation dose [weighted volume computed tomography dose index (CTDI_vol_) and effective dose], image noise, image quality, and polyp detection.

**Results:**

The mean effective dose of ULD‐CTC was significantly lower than that of LD‐CTC (0.98 and 2.69 mSv respectively, *P* < 0.0001) with an overall dose reduction of 63.2%. Image noise was comparable between ULD‐CTC and LD‐CTC (28.6 and 29.8 respectively, *P* = 0.09). There was no relevant difference when comparing image quality scores and polyp detection for both 2D and 3D images.

**Conclusion:**

ULD‐CTC allows to significantly reduce the radiation dose without meaningful image quality degradation compared to LD‐CTC.

1

**Key points**

- Ultra low‐dose CTC reduces radiation dose up to 63% compared to low‐dose CTC.
- SAFIRE reduces overall CTC image noise related to filtered back projection
- No relevant image quality deterioration is found when using ULD‐CTC protocol with SAFIRE compared to LD‐CTC protocol with filtered back projection.
- No significant differences in polyp detection were observed with ULD‐CTC compared to LD‐CTC

## 2 INTRODUCTION

Since its introduction in 1994,^1^ CT colonography (CTC) has progressively evolved to a validated examination for colorectal diseases. Evidence from the literature shows that the diagnostic performance for the detection of colorectal cancer and large polyps in symptomatic and asymptomatic individuals are similar to conventional colonoscopy and are largely superior to barium enema.^2^ Moreover, conventional colonoscopy is associated to increased anxiety, fear, and discomfort compared to CTC and, especially in elderly patients, it is burdened with the risk to have incomplete examination necessitating an alternative diagnostic method.^3^

The main disadvantage of CTC remains the use of ionizing radiation. This topic becomes of particular interest when CTC is proposed as a screening tool. Therefore, new strategies to keep the dose as low as reasonably achievable, without significantly sacrificing image quality, are strongly advisable.^4^, ^5^

Actually, the CTC dose is about one‐half of that from a conventional CT examination and in 2016 American College of Radiology recommended a radiation dose not exceeding 10 mSv^6^; the high natural contrast between the luminal gas, the soft tissue of the colonic wall or lesions, and residual tagged feces and fluids allow to use lower dose settings without compromising the core task of CTC, which is the detection of cancer and polyps.^7^

In this context, dose can be further reduced in CTC examinations by using automatic tube current modulation (ATCM), that automatically adjusts the x‐ray tube current (mAs) according to the size and attenuation characteristics of the body parts being examined and the scan plane. Many published studies demonstrated that ATCM leads to a significant reduction of radiation exposure in CTC.^7^, ^8^, ^9^

However, as the image noise is inversely proportional to the square root of the radiation dose, lower tube current results in increased noise‐related artifacts, thus worsening the quality of the images reconstructed with conventional filtered back projection (FBP) and decreasing radiologist's diagnostic confidence.^5^, ^9^

Many noise reduction methods based on image‐based iterative reconstruction algorithms have been developed for CT imaging and recent studies demonstrated the feasibility of low‐dose (LD) protocols beneficiating of comparable image quality and diagnostic performance.^10^, ^11^, ^12^, ^13^ Sinogram‐Affirmed Iterative Reconstruction (SAFIRE, Siemens Healthcare, Erlangen, Germany) is a hybrid iterative reconstruction technique incorporating a raw data‐based iterative reconstruction algorithm and an image space iteration algorithm.^14^, ^15^

Low‐dose and ultra‐low dose (ULD)‐CTC have been demonstrated to be feasible to reduce radiation dose maintaining a comparable image quality when using iterative reconstruction technique.^16^, ^17^, ^18^, ^19^ Based on these previous experiences, the aim of our study was to assess the radiation dose, image quality and performance in polyp detection of ultra low‐dose (ULD)‐CT colonography (CTC) obtained with the combined use of ATCM (CARE Dose 4D, Siemens Healthcare, Erlangen, Germany) with a quality reference mAs of 25 and SAFIRE, compared to low‐dose^1^ CTC acquired with CARE Dose 4D at quality reference mAs of 55 and reconstructed with FBP. Furthermore, we performed our analysis considering different patient sizes and colon segments.

## 3 MATERIALS AND METHODS

### 2.A Patients

All our procedures involving human subjects were in accordance with the ethical standards of the institutional research committee and with the 1964 Helsinki declaration and its later amendments or comparable ethic standards. The requirement for written informed consent was waived because the image data were retrospectively retrieved from routine CTC examinations.

A retrospective review of 89 clinically indicated CTC examinations consecutively performed in 13 months was conducted. The chief indication to CTC was incomplete colonoscopy. All CTCs were carried out with a 64‐detector detector CT scanner that can reconstruct 128 slices (Somatom Definition AS Plus, Siemens Healthcare, Erlangen, Germany), using a protocol that included ULD acquisition in the prone position and LD acquisition in the supine position. This CTC protocol was designed for our hospital after the introduction of CARE Dose 4D and SAFIRE, enabling the reduction of total radiation dose in CT examinations and CTC. As reported in the literature, the use of current modulation devices is strongly recommended and iterative reconstructions are preferred when performing CTC.^2^

Among the 89 patients, seven were excluded, one owing to the presence of motion artifacts, three because of large amount of residual colonic feces or fluids at sites of evaluation, and three in the reason of poorly distended colonic segments. Finally, a total of 82 patients (42 men and 40 women, with a mean age of 67.7 yr) were analyzed.

To account for patient size, as performed by Chang et al.,^20^ the anteroposterior (AP) diameter along the midline of each patient was measured on both supine and prone CT images at the level of the middle of the right kidney. The average prone‐supine AP diameter value was used to divide patients into five size groups, as follows: A. AP size of less than 22.0 cm; B. AP size of 22.1–24.0 cm; C. AP size of 24.1–26.0 cm; D. AP size of 26.1–28.0 cm; E. AP size larger than 28.0 cm.

### 2.B CTC preparation

To achieve adequate colonic cleansing, patients were instructed to maintain low residue diet and assume Macrogol (Movicol, Norgine Italia S.r.l., Milan, Italy) for three days before imaging. A same‐day oral tagging was obtained using Iopamidol (Gastromiro, Bracco Imaging, Milan, Italy). A balloon‐tipped silicone catheter was inserted via the rectum, and room air was manually introduced to maximum patient tolerance. Colonic insufflation was performed without any spasmolytic agent.

### 2.C CTC imaging protocol and image reconstruction

After adequate insufflation (as judged on scout images), image acquisition was performed in single breath holds to include the entire colon‐rectum, from the diaphragm to the greater trochanters of the femurs.

CTC acquisition parameters were: gantry rotation time 0.5 s, collimation 0.6‐mm, pitch 0.9.

The ULD prone acquisition was performed using a tube voltage of 120 kV and CARE Dose 4D with quality reference mAs of 25; images were reconstructed by SAFIRE, using a strength of 3.

The LD supine acquisition was performed using a tube voltage of 120 kV and CARE Dose 4D with quality reference mAs of 55; images were reconstructed by FBP.

All patients irrespective of their size were imaged with 120 kV; no 100 or 140 kV protocol was adopted for small or obese patients.

All images were reconstructed in a transverse orientation at a slice thickness of 1‐mm and an increment of 0.7‐mm and exported to our Picture and Archiving Communication System (PACS) and to a workstation equipped with the SyngoVia CT Colonography software (Siemens Healthcare, Erlangen, Germany) for the analysis.

### 2.D Radiation dose estimation

A radiology resident reviewed the structured DICOM dose reports automatically generated by the scanner at the end of the CT examinations and archived on PACS. The weighted volume computed tomography dose index (CTDI_vol_) and dose length product (DLP) values were recorded. The effective dose was obtained using DLP values and the normalized value of the conversion factor (E_DLP_; abdomen‐pelvis, 0.015 mSv/mGy·cm) proposed by the European Guidelines on quality Criteria,^14^, ^21^, ^22^ as follows: effective dose (mSv) = DLP × E_DLP_.

### 2.E Image noise measurements

A senior radiology resident calculated the image noise, by selecting a 100 mm^2^ region of interest (ROI) in the right psoas muscle on 2D axial images according to Chang et al.^5^ This ROI, not subject to partial volume averaging effects, was placed in the same location on prone and supine data sets by using “copy and paste” tool. Noise was defined as the mean standard deviation of psoas muscle attenuation.^5^, ^16^

### 2.F Image quality assessment

The image quality evaluation was performed by two experienced abdominal radiologists, with special practice in colorectal imaging and CTC, in consensus, after previewing ten test cases and agreeing how ratings would be determined. Images were presented to the radiologists by the study coordinator, after removing all DICOM headers as well as dose scanning or reconstruction reports.

### 2.G Two‐dimensional (2D) image quality assessment

Qualitative 2D image analysis was performed on both axial ULD and LD images using the CTC window setting (W: 1000 HU; L: 150 HU), for overall and each patient size group.

Image quality was evaluated at the rectosigmoid junction, the splenic flexure and the ileocecal valve, as performed by Flicek et al.,^16^ according to a five‐point scale, from 0 to 4 (0 for unacceptable, 1 for poor; 2 for suboptimal, 3 for average, and 4 for excellent).

Image noise was graded according to a five‐point scale, from 0 to 4 (0 for unacceptable, 1 for severe, 2 for moderate, 3 for mild, and 4 for minimum or absent).

Image sharpness was assessed by evaluating the aortic contour in the upper abdomen, according to a five‐point scale as well (0 for blurry edges, 1 for poorly defined edges, 2 for moderately unsharp edges, 3 for mildly unsharp edges, and 4 for very sharp edges).^16^

### 2.H Three‐dimensional (3D) image quality assessment

The ULD and LD endoluminal views were qualitatively evaluated for overall and each patient size group at the rectosigmoid junction, the splenic flexure and the ileocecal valve, considering sufficiently distended segments not covered by residual fluids. The electronic cleansing was not used in this study.

The amount of colonic mural surface irregularity was estimated on the basis of a four‐point scale (1 for severe with a characteristic cobblestone‐like appearance, 2 for moderate, 3 for mild, 4 for absent with a smooth surface of the colonic wall and sharp delineation of colonic folds).

Image quality was graded according to a five‐point scale, from 0 to 4 (0 for unacceptable, 1 for poor; 2 for suboptimal, 3 for average, and 4 for excellent).

### 2.I Polyp detection

*The same two readers involved in image quality assessment independently reviewed* LD‐CTC and ULD‐CTC images for polyp detection. The analysis was performed by the combined interpretation of 2D and 3D images, using CAD as concurrent reader. The number, site, and maximum diameter of all suspected lesions were recorded. To exactly locate each identified lesion, eight segments were considered: rectum, sigmoid colon, descending colon, splenic flexure, transverse colon, hepatic flexure, ascending colon, and cecum. The size of the polyps was measured in the 2D views and diminutive polyps (<6 mm) were not reported.^23^

### 2.J Statistical analysis

Data were tested for normality by using Shapiro–Wilk test. LD‐CTC and ULD‐CTC were compared in terms of radiation dose (CTDI_vol_ and effective dose), image noise, image quality, polyp detection, and size measurement by using Wilcoxon signed ranked test. The dose reduction rates were also calculated for each patient. For each of two protocols, differences among the five patient groups in terms of image noise and image quality were analyzed using the Kruskal–Wallis test. Concerning image quality assessment, a clinically relevant difference was considered to be present if a change in score of 1.0 or greater between ULD and LD protocol occurred, as performed by Chang.^20^

The interobserver agreement regarding polyp detection and size measurements was calculated through the use of Cohen's Kappa and Intraclass Correlation Coefficient (ICC), respectively.

Statistical significance was defined as *P* ≤ 0.05. Statistical analyses were performed using MedCalc 14.12.0 statistical software.

## 4 RESULTS

### 3.A Radiation dose evaluation

The mean CTDI_vol_ and effective dose values for LD and ULD protocols and their reduction rates are described in Tables 1 and 2, respectively.

**Table 1:** Mean CTDI_vol_ and effective dose values

Group	Mean CTDI_vol_ (mGy)			Mean effective dose (mSv)		
	Low dose	Ultra‐low dose	*P* value^a^	Low dose	Ultra‐low dose	*P* value^a^
A n.14	3.10 (0.53)	1.05 (0.13)	0.001	2.05 (0.43)	0.73 (0.13)	0.001
B n.18	3.51 (0.63)	1.19 (0.15)	<0.0001	2.43 (0.46)	0.88 (0.14)	<0.0001
C n.19	3.75 (0.72)	1.30 (0.24)	<0.0001	2.61 (0.45)	0.97 (0.19)	<0.0001
D n.17	4.23 (0.81)	1.38 (0.15)	0.001	2.96 (0.77)	1.02 (0.12)	<0.0001
E n.14	4.81 (1.19)	1.70 (0.63)	0.001	3.45 (0.83)	1.31 (0.49)	0.001
Overall	3.87 (0.96)	1.32 (0.36)	<0.0001	2.69 (0.74)	0.98 (0.30)	<0.0001

Values in parentheses are the standard deviation.

n, number of patients; CTDI_vol_, weighted volume computed tomography dose index.

a Wilcoxon signed ranked test.

**Table 2:** Mean CTDI_vol_ and effective dose values reduction rates

Group	CTDI_vol_ reduction rate (LD vs ULD)	Effective Dose reduction rate (LD vs ULD)
A	65.4%	63.8%
B	65.6%	63.3%
C	64.9%	62.4%
D	66.7%	64.3%
E	64.9%	62.5%
Overall	65.5%	63.2%

LD: low dose; ULD: ultra low dose; CTDI_vol_, weighted volume computed tomography dose index.

**Figure 1:**
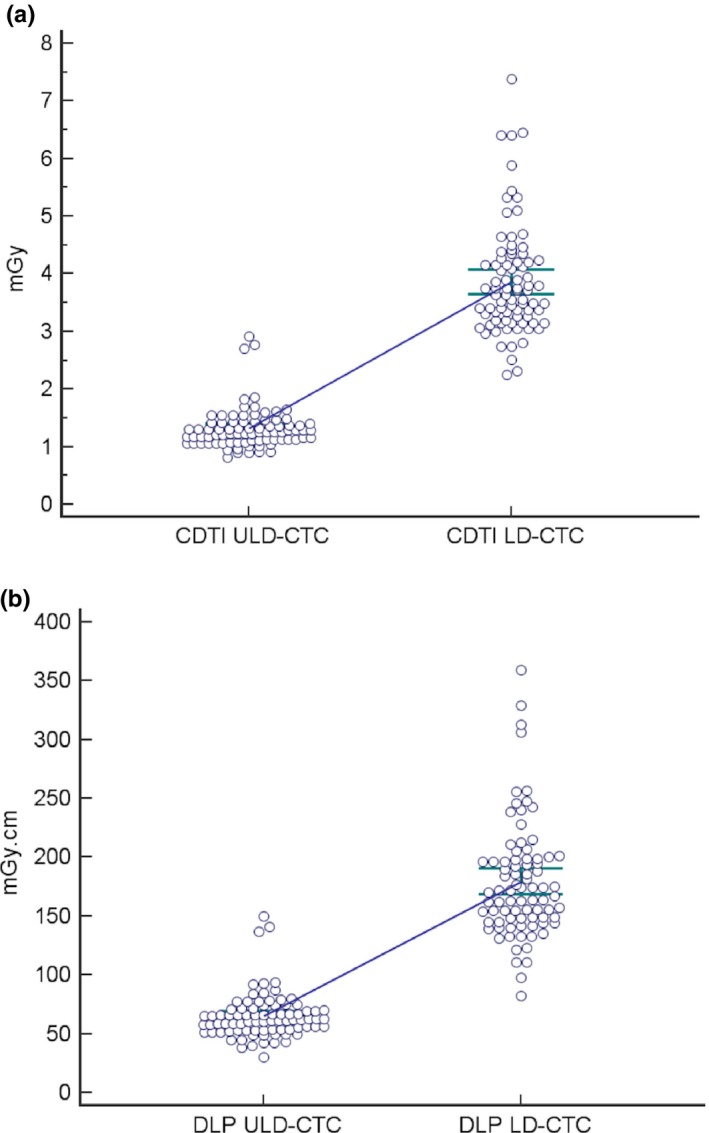
Plots of CTDI_vol_ (a) and Effective Dose (b) values obtained with LD‐CTC and ULD‐CTC.

In detail, CTDI_vol_ and effective dose values significantly decreased when using the ULD protocol compared to LD protocol (*P* < 0.0001). Furthermore, the overall mean effective dose of the ULD protocol (0.98 ± 0.30 mSv) was significantly lower than that of the LD protocol (2.69 ± 0.74 mSv) (*P *< 0.0001; Fig. 1). Concerning the overall dose reduction when using the ULD protocol, we observed a 65.5% and a 63.2% for CTDI_vol_ and effective dose, respectively. Successful dose savings were achieved for all five patient groups independently from patient size (Table 2).

### 3.B Image noise measurements

Results of image noise measurements are shown in Table 3.

**Table 3:** Comparison of image noise measurements

Group	Image noise		*P* value^a^
	Low dose	Ultra‐low dose	
A	23.7 (6.5)	23.9 (5.3)	0.75
B	27.6 (5.3)	26.3 (5.9)	0.24
C	30.5 (4.2)	29.4 (3.8)	0.74
D	31.6 (4.6)	29.5 (3.5)	0.18
E	35.6 (4.9)	34.2 (4.9)	0.3
Overall	29.8 (6.2)	28.6 (5.7)	0.09

**Figure 2:**
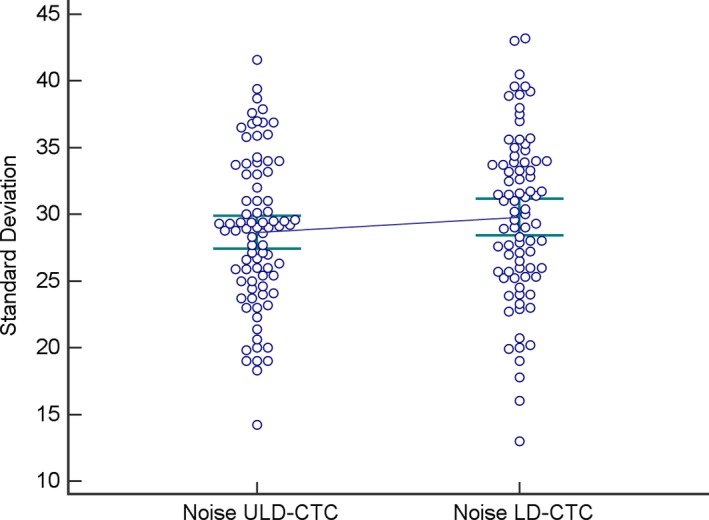
Plots of objective noise values obtained with LD‐CTC and ULD‐CTC.

Considering the overall results, ULD protocol showed lower image noise (28.6 ± 5.7) compared to LD protocol (29.8 ± 6.2), but the difference between two protocols was not statistically significant (*P* = 0.09; Fig. 2). No significant differences between ULD and LD protocol were found when considering each one of the five groups.

Furthermore, concerning the pairwise comparison among five groups, the mean image noise was independent from patient size except when comparing A and B groups with and E group for both protocols (*P* < 0.0001 and *P* = 0.001, respectively, ULD protocol; *P* = 0.014 and *P* < 0.0001, respectively, LD protocol).

### 3.C Image quality assessment

The results of the 2D image quality evaluation are shown in Table 4.

**Table 4:** Results of 2D image quality assessment

Group	Image quality rectosigmoid junction		*P* value^a^	Image quality splenic flexure		*P* value^a^	Image quality ileocecal valve		*P* value^a^	Image noise		*P* value^a^	Aortic sharpness		*P* value^a^
	LD	ULD		LD	ULD		LD	ULD		LD	ULD		LD	ULD	
A	3.71 (0.19)	3.79 (0.16)	0.317	3.54 (0.18)	3.38 (0.21)	0.317	3.67 (0.14)	3.42 (0.23)	0.083	3.36 (0.17)	2.42 (0.14)	<0.0001	3.21 (0.19)	2.36 (0.13)	0.001
B	3.89 (0.08)	3.94 (0.06)	0.317	3.67 (0.14)	3.61 (0.14)	0.317	3.88 (0.08)	3.65 (0.15)	0.046	3.28 (0.14)	2.39 (0.11)	<0.0001	3.33 (0.14)	2.56 (0.12)	<0.00001
C	3.89 (0.07)	3.79 (0.12)	0.157	3.58 (0.12)	3.63 (0.14)	0.564	3.71 (0.11)	3.53 (0.15)	0.083	3.10 (0.10)	2.21 (0.10)	<0.0001	3.11 (0.13)	2.26 (0.10)	<0.00001
D	3.76 (0.11)	3.71 (0.11)	0.317	3.65 (0.12)	3.59 (0.12)	0.317	3.82 (0.10)	3.53 (0.15)	0.059	3.35 (0.12)	2.47 (0.12)	<0.0001	3.35 (0.12)	2.59 (0.12)	<0.00001
E	3.85 (0.10)	3.77 (0.17)	0.317	3.57 (0.14)	3.64 (0.13)	0.317	3.71 (0.16)	3.29 (0.22)	0.034	3.29 (0.13)	2.36 (0.13)	<0.0001	3.14 (0.14)	2.57 (0.20)	0.005
Overall	3.83 (0.05)	3.80 (0.05)	0.414	3.60 (0.06)	3.58 (0.07)	0.527	3.77 (0.46)	3.49 (0.68)	<0.0001	3.27 (0.06)	2.37 (0.05)	<0.0001	3.23 (0.06)	2.46 (0.06)	<0.0001

Values in parentheses are standard deviation.

LD: low dose; ULD: ultra low dose.

a Wilcoxon test.

**Figure 3:**
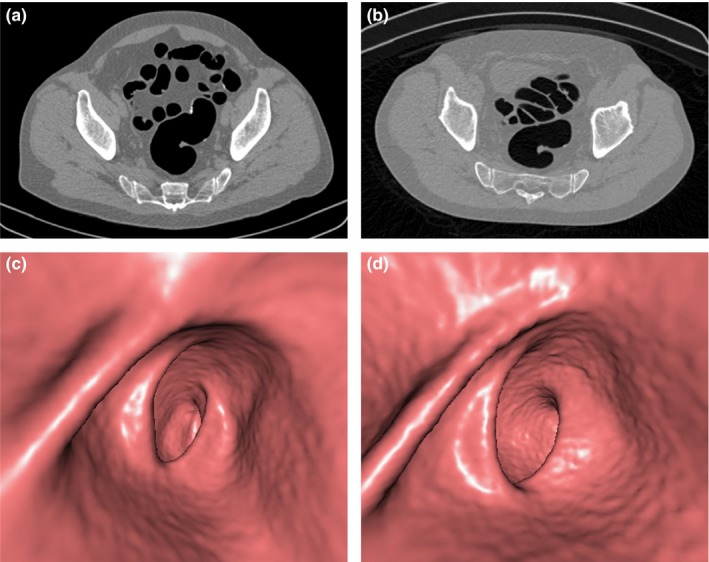
Axial two‐dimensional (2D) computed tomography images (a,b) and three‐dimensional (3D) endoluminal reconstructions (c,d) of the rectosigmoid junction in a 69‐yr‐old, class D, man after incomplete colonoscopy. Both 2D LD (a) and ultra‐low dose (ULD) (b) images were rated to have an average image quality, with mild image noise. Similarly, both 3D low‐dose (LD) (c) and ULD (d) images were judged to have an average image quality, despite a slight increase in mural surface irregularity for the ULD protocol (d).

**Figure 4:**
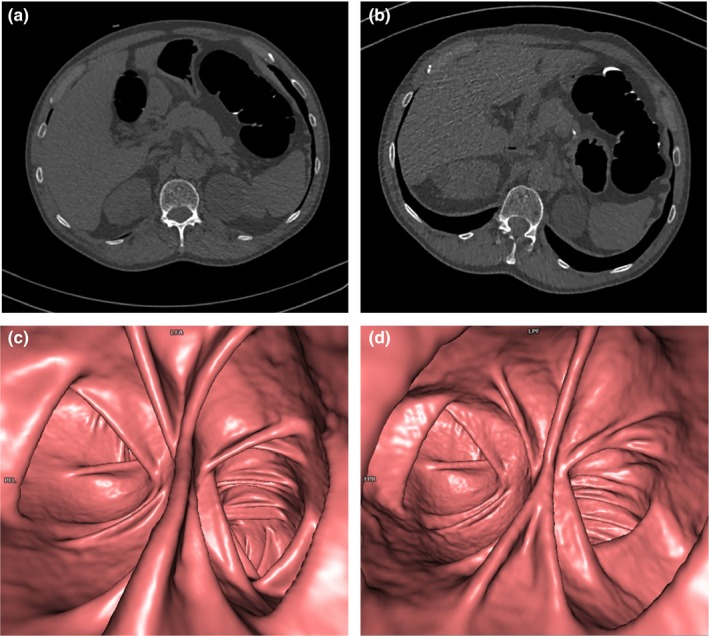
Axial two‐dimensional (2D) computed tomography images (a,b) and three‐dimensional (3D) endoluminal reconstructions (c,d) of the splenic flexure in a 71‐yr‐old, class B, man after incomplete colonoscopy. Both 2D low‐dose (a) and ultra‐low dose (ULD) (b) images were rated to have comparable overall image quality, despite a slight increase of the perceived noise and aortic contour blurriness of the ULD protocol. Similarly, no significant differences were found between overall 3D image quality scores of ULD and LD acquisitions, despite a slight increase of the mural surface irregularity of the ULD protocol (d).

**Figure 5:**
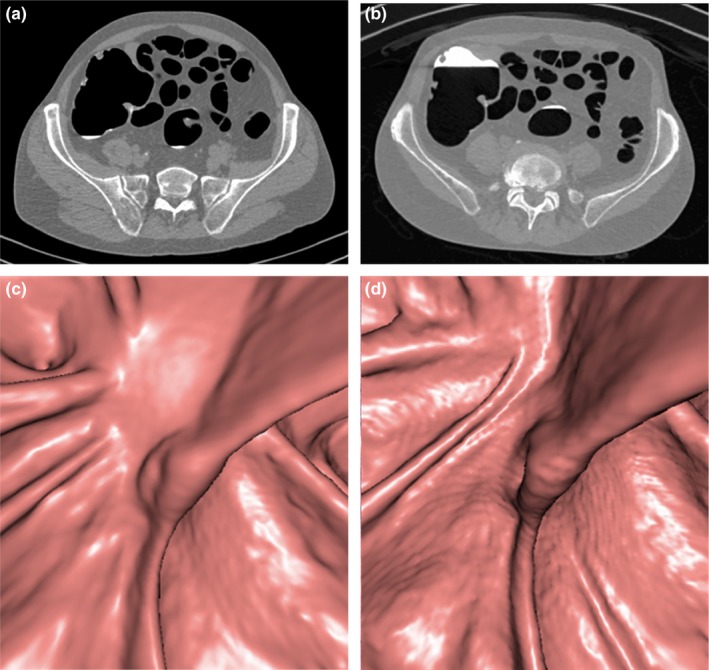
Axial two‐dimensional (2D) computed tomography images (a,b) and three‐dimensional 3D endoluminal reconstructions (c,d) at the level of the ileocecal valve in a 67‐yr‐old, class D, man with a history of colonic polyps and incomplete colonoscopy. Low‐dose (a) and ultra‐low dose (ULD) (b) 2D images were deemed to have equivalent overall quality, but there was a less subjective noise for the ULD acquisition with sinogram‐affirmed iterative reconstruction (b). No significant differences were found in overall 3D image quality, although there was a mild increase of mural surface irregularity for the ULD acquisition (d).

Overall, 2D image quality of LD and ULD protocols was quite similar at the rectosigmoid junction (3.83 and 3.80, respectively) and at the splenic flexure (3.60 and 3.58, respectively); they differed at the ileocecal valve (3.77 and 3.49, *P *< 0.0001). Anyway, when considering the mean score in all patient size groups and at each site of evaluation, there was no difference equal or greater to1.0 when comparing ULD and LD protocols (Figs. 3, 4, 5).

Although image noise and aortic sharpness scores of ULD protocol were lower than those of LD protocol, differences resulted to be not significant.

The results of the 3D image quality evaluation are shown in Table 5.

**Table 5:** Two‐dimensional image quality assessment: comparison of image quality and mural surface irregularity scores

Group	Image quality		*P* value^a^	Irregularity		*P* value^a^
	LD	ULD		LD	ULD	
Rectosigmoid junction						
A	3.79 (0.11)	3.29 (0.13)	0.008	3.79 (0.11)	3.29 (0.13)	0.008
B	3.94 (0.06)	3.33 (0.11)	0.001	3.94 (0.06)	3.22 (0.13)	<0.0001
C	3.89 (0.07)	3.37 (0.11)	0.002	3.89 (0.07)	3.32 (0.11)	0.0001
D	3.94 (0.06)	3.11 (0.08)	<0.0001	3.94 (0.06)	3.12 (0.08)	<0.0001
E	3.86 (0.10)	3.00 (0.10)	0.001	3.86 (0.10)	3.00 (0.10)	0.0001
Overall	3.90 (0.03)	3.23 (0.05)	<0.0001	3.89 (0.03)	3.20 (0.05)	<0.0001
Splenic flexure						
B	3.89 (0.08)	3.44 (0.12)	0.005	3.83 (0.09)	3.50 (0.12)	0.014
C	3.79 (0.12)	3.26 (0.13)	0.002	3.79 (0.12)	3.42 (0.14)	0.008
D	3.94 (0.06)	3.12 (0.08)	<0.0001	3.82 (0.10)	3.06 (0.10)	<0.0001
E	3.86 (0.10)	3.14 (0.10)	0.002	3.93 (0.07)	3.14 (0.10)	0.001
Overall	3.84 (0.04)	3.28 (0.06)	<0.0001	3.83 (0.05)	3.35 (0.06)	<0.0001
Ileocecal valve						
A	3.70 (0.15)	3.30 (0.15)	0.102	3.80 (0.13)	3.30 (0.15)	0.059
B	3.81 (0.10)	3.06 (0.11)	0.001	3.94 (0.06)	3.25 (0.11)	0.001
C	3.81 (0.14)	3.19 (0.14)	0.002	3.81 (0.14)	3.38 (0.15)	0.008
D	3.81 (0.10)	3.00 (0.16)	0.001	3.88 (0.09)	3.00 (0.16)	<0.0001
E	3.75 (0.13)	3.00 (0.12)	0.003	3.83 (0.11)	3.08 (0.08)	0.007
Overall	3.79 (0.06)	3.10 (0.06)	<0.0001	3.87 (0.05)	3.20 (0.06)	<0.0001

Values in parentheses are standard deviation.

LD: low dose; ULD: ultra low dose.

a Wilcoxon test.

Overall, 3D image quality for LD and ULD protocols was respectively 3.90/3.23 (*P* < 0.0001) at the rectosigmoid junction, 3.84/3.28 (*P* < 0.0001) at the splenic flexure, and 3.79/3.10 (*P* < 0.0001) at the ileocecal valve. More in detail, in all patient size groups and at each site of evaluation, the image quality scores of the ULD protocol were lower than those of the LD protocol, with a maximum difference of 0.86 at rectosigmoid junction for larger patients of group E. The colonic wall irregularity when using the ULD protocol was higher compared to LD protocol, but differences did not exceed 0.69. Furthermore, concerning the mean score in all patient size groups and at each site of evaluation, there was no difference equal or greater to 1.0 when comparing ULD and LD protocols as well as for the 2D image quality assessment (Figs. 3, 4, 5).

### 3.D Polyp detection

Of 82 patients, 36 had a complete colonoscopy or surgery performed within one month from CTC; nine polyps with a diameter between 6 and 9 mm, and seven polyps ≥1 cm were removed. The detection rate of LD‐CTC and ULD‐CTC was 14/16 (88%). Both LD‐CTC and ULD‐CTC missed two small polyps, one (6 mm in size) abutting the ileocecal valve, and one (7 mm in size) located in the sigmoid colon and surrounded by multiple diverticula showing fecal impaction. Both these missed lesions resulted to be hyperplastic at histology.

No false‐positive cases occurred in our study population.

When comparing LD‐CTC and ULD‐CTC, no significant differences in terms of detection rate and size measurement (*P* > 0.05) were observed. The interobserver agreement between the two readers was good (0.77, Cohen's Kappa) and excellent (0.91, ICC) for the detection rate and size measurement, respectively.

## 5 DISCUSSION

CTC is a reliable examination to detect colorectal polyps and cancers. Although the CTC radiation dose is about half than that of conventional CT examinations, the main concern regarding the use of this technique relies on the x‐ray exposure. The mean effective dose reported in our study when using the LD protocol was 2.69 mSv, a value that is in agreement with previous studies.^24^, ^25^ In the light of promising results provided by recently developed iterative reconstruction methods, several studies investigated the possibility to further reduce the radiation dose showing an appreciable image noise reduction without a noticeable loss of image quality.^16^, ^17^, ^18^, ^19^

In this context, our results validated the hypothesis that by using the automated tube current modulation and SAFIRE, radiation dose of CTC could be further reduced below 1 mSv, without substantially affecting diagnostic image quality and independently from patient sizes and the colon segments.

More in detail, with a quality reference mAs of 25, a further radiation dose reduction of 63.2% (0.98 mSv) was found to be possible (compared to the LD protocol), without significant impairment of image quality. A significant decrease in CTDI_vol_ and effective dose values was noted in all patient groups. Patient size did not significantly affect the percentage of dose savings, probably because automated tube current modulation was used on both ULD and LD protocols, leading to a patient‐tailored x‐ray exposure.

On quantitative analysis, image noise values of the ULD protocol resulted to be lower than those of the LD protocol with FBP in all patient size groups. In this way, the good performance in decreasing noise independently from patient body habitus, encourages its wide clinical use including larger patients.

Conversely, when considering 2D images evaluation, quality scores of ULD protocol resulted to be slightly lower than those of LD protocol for each patient group at each site of evaluation and significantly lower (but not exceeding 1.0 in terms of change of score) at the ileocecal valve. In our opinion, these results were mainly due to the perceived increased noise and coarsening of the noise texture with SAFIRE. Moreover, when considering the aortic contour, it was judged as moderately unsharp on ULD protocol and mildly unsharp on LD protocol with significant differences between the two protocols. As a matter of fact, the processing of iterative reconstruction may decrease image sharpness and provide a different image texture, resulting in a blurry or blotchy aspect. The smoothing appearance of the 2D images reconstructed with SAFIRE may be somewhat unappealing to radiologists who are not familiar to it and therefore it may reduce subjective image quality scores.

A SAFIRE strength of 3 was used in our study. Five different strengths are offered with SAFIRE (S1, S2, S3, S4, S5). The level of noise reduction and noise texture will change depending on the chosen strength, with S1 being noisier and S5 being smoother. In detail, S1 setting leads to sharp and well‐demarcated organ contours, but images are coarse, with many artifacts that can affect their interpretation; S5 images have a decreased noise, but they are more blurred and blotchy in appearance. S3 provides a good balance between image noise and image texture, and it resulted to be the preferred SAFIRE strength for radiologists when evaluating subjective abdominal CT imaging quality.^26^, ^27^

Similarly to 2D evaluation, in all patient groups, image quality of 3D ULD images was lower than that of LD images, with a mild increase of the observed colonic wall irregularity. Anyway, 2D and 3D image quality score differences between ULD and LD protocols resulted to be not relevant (less than 1.0), thus suggesting the suitability of ULD technique for daily CTC examinations.

The slightly lower image quality of 2D and 3D ULD‐CTC did not interfere with polyp identification in our series. Actually, the ULD‐CTC showed a good performance in polyp detection, without significant differences when compared with LD‐CTC. In detail, all seven large polyps and seven out nine small polyps were correctly identified on both protocols, despite the perceived increased noise and colonic wall irregularity with ULD‐CTC.

Our results agree with findings of recently published works. Fletcher et al.^28^ demonstrated a comparable image quality for colonic evaluation between full‐dose CTC images and half‐dose CTC images reconstructed with SAFIRE by using a dual‐source single‐tube reconstruction method.^28^

Although we analyzed a protocol by a single vendor, using the ATCM and SAFIRE, other studies suggest that ULD‐CTC is feasible to reduce radiation dose maintaining a comparable image quality when using iterative reconstruction technique.^16^, ^17^, ^18^, ^19^

More in detail, Flicek et al. 16 showed that the radiation dose for CTC may be 50% reduced below currently accepted low‐dose techniques without significantly sacrificing image quality when ASIR is used. Millerd et al. 17 demonstrated that radiation dose in CTC using model‐based iterative reconstruction (MBIR) may be decreased by 60% while maintaining overall image quality and reducing image noise. Lubner et al.^19^ used iterative reconstruction methods, including MBIR, in 40 subjects undergoing a sub‐mSv CTC acquisition and found that this technique may reduce image noise and improve image quality when compared to reduced dose images reconstructed with FBP. Lambert et al.^18^ showed that advanced hybrid iterative reconstruction technique is a feasible method to decrease the radiation dose from both supine and prone CTC below 1 mSv while preserving a good image quality.

When considering diagnostic performance in polyp detection, our results are in line with those of other published works. Lubner et al.^24^ found that polyp conspicuity was similar on standard dose images compared to reduced dose images reconstructed with MBIR. Lambert et al.^29^ demonstrated that iterative reconstructions are suitable for sub‐milliSievert ultralow‐dose CTC without sacrificing diagnostic performance of the study. Shin et al.^30^ stated that the per‐polyp sensitivity of one‐mSv CTC can be improved with the application of iterative reconstruction algorithms, when compared with the standard FBP algorithms.

One limitation of our study is that it was impossible to totally blind readers to the images being analyzed (ULD prone vs LD supine), particularly with 2D images in which patient position is easily recognizable by the tagged fluid disposition. The standard acquisition protocol for CTC consists in a combination of prone and supine positions.^31^ The variability in the degree of luminal distension in the supine and prone positions could have an unpredictable effect on the overall image quality and noise. It would be better to compare ULD and LD images in the same prone or supine position for the same individual, but this was not possible owing to the retrospective design of the study. When we defined our CTC protocol, we applied the ULD technique to the prone dataset prior to incorporate it to both scans, having the patient as his or her own control and reducing the risk of a nondiagnostic CTC.^5^ Further prospective studies could be useful to control the variable of patient decubitus, for example randomly assigning ULD protocol either to prone or supine acquisition.

Another limitation is that no comparison of image quality was done between ULD with FBP and SAFIRE; anyway, the added value of iterative reconstruction is widely demonstrated in previous studies on abdominal CT imaging.^5^, ^16^, ^30^, ^32^

In conclusion, this study demonstrates that the radiation dose for CTC can be further reduced up to 63% to a sub‐mSv acquisition by modulating the tube current. When applying SAFIRE to ULD images, it is possible to reduce image noise without a significant impact on image quality and polyp detection.

## AUTHORS’ CONTRIBUTIONS

All authors were involved in patient management and wrote the report. Written consent to publication was obtained.

## CONFLICT OF INTEREST

The authors declare that they have no conflict of interest.
